# *Bordetella* Dermonecrotic Toxin Is a Neurotropic Virulence Factor That Uses Ca_V_3.1 as the Cell Surface Receptor

**DOI:** 10.1128/mBio.03146-19

**Published:** 2020-03-24

**Authors:** Shihono Teruya, Yukihiro Hiramatsu, Keiji Nakamura, Aya Fukui-Miyazaki, Kentaro Tsukamoto, Noriko Shinoda, Daisuke Motooka, Shota Nakamura, Keisuke Ishigaki, Naoaki Shinzawa, Takashi Nishida, Fuminori Sugihara, Yusuke Maeda, Yasuhiko Horiguchi

**Affiliations:** a Department of Molecular Bacteriology, Research Institute for Microbial Diseases, Osaka University, Suita, Osaka, Japan; b Department of Microbiology, Fujita Health University School of Medicine, Toyoake, Aichi, Japan; c Department of Infection Metagenomics, Research Institute for Microbial Diseases, Osaka University, Suita, Osaka, Japan; d Central Instrumentation Laboratory, Research Institute for Microbial Diseases, Osaka University, Suita, Osaka, Japan; e Department of Molecular Virology, Research Institute for Microbial Diseases, Osaka University, Suita, Osaka, Japan; Medical College of Wisconsin; GSK Vaccines

**Keywords:** pertussis, dermonecrotic toxin, receptor, encephalopathy

## Abstract

Dermonecrotic toxin (DNT) is one of the representative toxins produced by Bordetella pertussis, but its role in pertussis, B. pertussis infection, remains unknown. In this study, we identified the T-type voltage-gated Ca^2+^ channel Ca_V_3.1 as the DNT receptor by CRISPR-Cas9-based genome-wide screening. As Ca_V_3.1 is highly expressed in the nervous system, the neurotoxicity of DNT was examined. DNT affected cultured neural cells and caused flaccid paralysis in mice after intracerebral injection. No neurological symptoms were observed by intracerebral injection with the other major virulence factors of the organisms, pertussis toxin and adenylate cyclase toxin. These results indicate that DNT has aspects of the neurotropic virulence factor of B. pertussis. The possibility of the involvement of DNT in encephalopathy, which is a complication of pertussis, is also discussed.

## Abstract (precis)

Bordetella pertussis, which causes pertussis, a contagious respiratory disease, produces three major protein toxins, pertussis toxin, adenylate cyclase toxin, and dermonecrotic toxin (DNT), for which molecular actions have been elucidated. The former two toxins are known to be involved in the emergence of some clinical symptoms and/or contribute to the establishment of bacterial infection. In contrast, the role of DNT in pertussis remains unclear. Our study shows that DNT affects neural cells through specific binding to the T-type voltage-gated Ca^2+^ channel that is highly expressed in the central nervous system and leads to neurological disorders in mice after intracerebral injection. These data raise the possibility of DNT as an etiological agent for pertussis encephalopathy, a severe complication of B. pertussis infection.

## INTRODUCTION

Bordetella pertussis causes pertussis (whooping cough), a highly contagious respiratory disease that is characterized by a wide range of clinical manifestations, including bronchopneumonia, hypoglycemia, leukocytosis, and paroxysmal coughing. The disease also infrequently develops encephalopathy as a sequela that may cause death or permanent neurological disorders (1–7). Although the molecular activities of B. pertussis virulence factors have been analyzed in depth, the pathogenesis of pertussis is not well understood (8–10). The organism produces three representative protein toxins, pertussis toxin (PT), adenylate cyclase toxin (ACT), and dermonecrotic toxin (DNT). PT catalyzes ADP ribosylation on the heterotrimeric GTPases of the Gαi subfamily via the enzymatically active domain and can multivalently bind to sialic acid-containing glycoproteins via its receptor-binding oligomer (8, 11). These toxic actions are considered to be related to host immune modulations and some clinical symptoms such as hypoglycemia and leukocytosis (8, 9). ACT increases the level of intracellular cAMP in target cells to a supraphysiological level, which results in immunomodulation, including the marked production of cytokines and dysfunction of immunocompetent myeloid cells (8, 9, 12). Previous studies using animal models reported that these two toxins function in the establishment of bacterial infection by altering the inflammatory responses of hosts (8, 9, 12).

In contrast, nothing is known about the role of DNT in pertussis. DNT is a single-chain polypeptide of 1,464 amino acids. The N-terminal 30-amino-acid region is responsible for binding to target cells via an unknown receptor, and the ∼300-amino-acid C terminus carries an enzymatically active domain with transglutaminase activity that activates the small GTPases of the Rho family through deamidation or polyamination (13–17). DNT of Bordetella bronchiseptica, which is virtually identical to that of B. pertussis (see Fig. S1 in the supplemental material), is known to cause turbinate atrophy by inhibiting osteoblastic differentiation in atrophic rhinitis, B. bronchiseptica infection of pigs (18–20). However, the role of DNT in B. pertussis infection has not been elucidated. In pertussis, unlike atrophic rhinitis, no pathological abnormality in bone tissues has been reported. Moreover, possible target organs/tissues other than bone tissues have not been explored.

10.1128/mBio.03146-19.3 FIG S1 Comparison of biological and biochemical properties of DNT from B. pertussis and B. bronchiseptica. In this study, we mainly used the recombinant protein of B. bronchiseptica DNT, which was previously well characterized (see reference 6 in Text S2 in the supplemental material). Recombinant DNT of B. pertussis was used for animal experiments. DNTs from B. bronchiseptica and B. pertussis had been considered to be substantially identical without experimental evidence. The following results demonstrate that both types of DNT similarly affect target cells via transglutaminating activity on Rho proteins. Thus, both types of DNT are referred to as DNT in the text, unless otherwise specified. (A) Amino acid sequence alignment of the receptor-binding regions of B. bronchiseptica DNT (BB-DNT) and B. pertussis DNT (BP-DNT). BB-DNT and BP-DNT share 99.0% amino acid sequence identity of the full-length molecules. Asterisks indicate identical amino acid residues. The receptor-binding domain (see reference 7 in Text S2) is in green. (B to D) SDS-PAGE and immunoblotting of BB-DNT, BP-DNT, and MC3T3-E1 cells treated with DNT. (B) DNT samples were stained with Coomassie brilliant blue R250 after SDS-PAGE. *, molecular weight markers. (C) For immunoblotting, DNT was probed with an anti-DNT polyclonal antibody (pAb) and anti-DNT monoclonal antibodies 1A3 and 2B3. Note that BB-DNT-specific 2B3 (see reference 2 in Text S2) did not recognize BP-DNT preparations because of the substitution of Ser^53^ for Gly^53^. (D) MC3T3-E1 cells were treated with DNT and subjected to immunoblotting for the DNT-catalyzed deamidation of Rho with anti-Rho^63E^ polyclonal antibody or anti-β-tubulin. W, wild type; C, DNT_C1305A_ (an enzymatically inactive derivative of DNT). (E) Microscopic images of MC3T3-E1 cells exposed to BB-DNT or BP-DNT at the indicated concentrations for 16 h. Bar, 100 μm. Note that the characteristic morphological changes of the cells were observed after treatment with both types of DNT at the indicated range of concentrations (50 to 500 ng/ml). Download FIG S1, EPS file, 0.6 MB. Copyright © 2020 Teruya et al. 2020 Teruya et al. This content is distributed under the terms of the Creative Commons Attribution 4.0 International license.

In this study, we aimed at identifying a receptor(s) for DNT and, based on its tissue distribution, searched for potential target organs/tissues. Our results demonstrate that DNT recognizes the T-type voltage-gated Ca^2+^ channels Ca_V_3.1 and Ca_V_3.2 as cell surface receptors. According to public databases, Ca_V_3.1 is dominantly expressed in the nervous system. Indeed, the toxin affected cultured neural cells that expressed Ca_V_3.1. In addition, intracerebral injection of DNT caused flaccid paralysis in mice. We concluded that DNT has aspects of the neurotropic virulence factor of B. pertussis. The possibility of its involvement in pertussis encephalopathy is also discussed.

## RESULTS

### CRISPR-Cas9 screening for DNT receptors.

**FIG 1:**
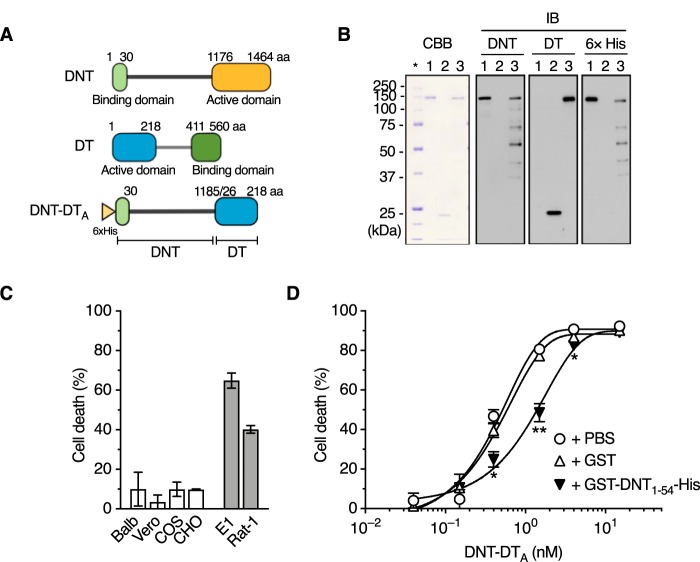
Construction of DNT-DT_A_. (A) Schematic representation of DNT-DT_A_. aa, amino acids. (B) SDS-PAGE and immunoblotting of the purified preparations of DNT, DT_A_, and DNT-DT_A_. DNT (lane 1), DT_A_ (lane 2), and DNT-DT_A_ (lane 3) were applied at 0.5 μg/lane for Coomassie brilliant blue R250 (CBB) staining and at 0.1 μg/lane, 0.2 μg/lane, and 0.2 μg/lane, respectively, for immunoblotting (IB). The samples were probed with an anti-DNT polyclonal antibody, an anti-DT polyclonal antibody, and an anti-His tag antibody. The asterisk indicates the lane for marker proteins. Note that DNT-DT_A_ was recognized by anti-DNT and anti-DT antibodies. (C) Sensitivity of cultured cells to DNT-DT_A_. DNT-resistant (white bars, Balb3T3, Vero, COS7, and CHO-K1) and -sensitive (gray bars, MC3T3-E1 and Rat-1) cells were incubated with DNT-DT_A_ for 16 h, and the rate of cell death was measured. (D) Competitive inhibition of cytotoxicity of DNT-DT_A_ with GST (glutathione *S*-transferase)-DNT_1–54_-His. MC3T3-E1 cells were treated with DNT-DT_A_ in the presence of 400 nM GST or GST-DNT_1–54_-His, and the rate of cell death was measured. *, *P < *0.01; **, *P* < 0.001 (compared to PBS). Plotted data represent means ± standard errors of the means (SEM) (*n* = 3) (C and D).

**FIG 2:**
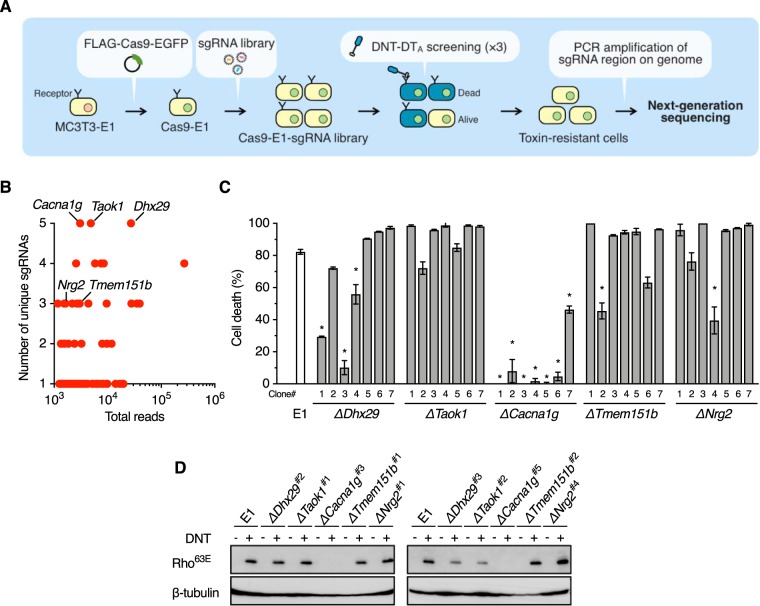
Genome-wide screening for DNT receptors by the CRISPR-Cas9 system. (A) Procedures for genome-wide screening for the DNT receptor(s). (B) Genes identified after screening. The *y* and *x* axes represent the numbers of unique sgRNAs and sgRNA sequence reads for each identified gene, respectively. (C) Sensitivities of the candidate-gene knockout clones of MC3T3-El to DNT-DT_A_. Seven knockout clones were selected for each gene and treated with DNT-DT_A_, and the rate of cell death was evaluated. Plotted data represent means ± SEM (*n* = 3). *, *P < *0.0001 (compared to MC3T3-E1 parental cells [E1]). (D) DNT-induced deamidation of Rho in the knockout clones of MC3T3-E1. Two of the seven clones shown in panel C for each gene were further selected and treated with DNT. The numbers of selected clones are shown after the gene names.

In order to identify a DNT receptor, we adopted genome-wide screening with the CRISPR-Cas9 system, which is a powerful technique to identify desired genes. However, DNT is unsuitable as a probe for high-throughput screening because the enzyme action of DNT, which does not cause cell death, is not readily detected. We therefore generated DNT-DT_A_, which consists of the N-terminal fragment of DNT, including the receptor-binding domain and the translocation domain (DNT_2–1185_) (21), and the active domain of diphtheria toxin (DT_26–218_) (Fig. 1A and B). DNT-DT_A_ caused the death of DNT-sensitive MC3T3-E1 and Rat-1 cells but not resistant cells (22, 23) (Fig. 1C). The cytotoxicity of DNT-DT_A_ was inhibited in the presence of the binding-domain-containing DNT_1–54_, suggesting that DNT-DT_A_ intoxicates the cells by binding to the receptor for DNT (Fig. 1D). Using DNT-DT_A_ as the probe, we carried out screening (Fig. 2A). MC3T3-E1 cells that stably expressed Cas9 were transduced with the lentiviral library of single guide RNAs (sgRNAs) targeting 19,150 genes (5 unique sgRNAs for each gene). After three rounds of screening with DNT-DT_A_, sgRNA regions integrated into the genomic DNA of DNT-DT_A_-resistant cells were sequenced, and targeted genes were identified (Fig. 2B and Table S3). From the identified genes, we picked up *Dhx29*, *Taok1*, and *Cacna1g*, on the basis of the number of unique sgRNAs, and genes encoding membrane proteins, *Tmem151b* and *Nrg2*, and generated 7 clones of MC3T3-E1 mutants for each gene for further analyses. Sensitivity to DNT-DT_A_ was markedly reduced or abolished in Δ*Cacna1g* cells, whereas almost all of the other mutant cells were sensitive, but some clones exhibited reduced sensitivity (Fig. 2C). Therefore, we arbitrarily selected 2 of 7 clones for each mutant and further examined their sensitivity to DNT (Fig. 2D). As judged by the deamidation of intracellular Rho (Rho^63E^), Δ*Cacna1g* cells were confirmed to be resistant to DNT, demonstrating that *Cacna1g* is involved in the intoxication process of the toxin.

### Ca_V_3.1 serves as the DNT receptor.

**FIG 3:**
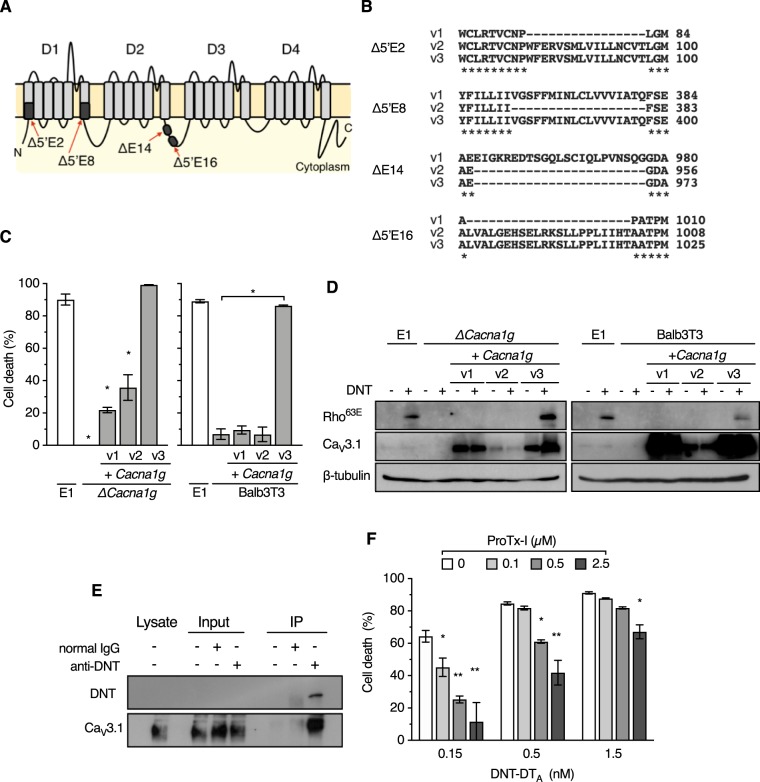
Identification of Ca_V_3.1 as a receptor for DNT. (A) Schematic illustration of Ca_V_3.1 consisting of four distinct domains (D1 to D4). Missing regions by alternative splicing in the variants are indicated by black boxes. Each region is designated according to the designations in a previous study (24). (B) Amino acid sequence alignments of the spliced regions of v1, v2, and v3. Dashes indicate missing regions of the splice variants. Asterisks indicate conserved amino acid residues. (C) Ectopic expression of *Cacna1g* v3 restores the sensitivity of MC3T3-E1/Δ*Cacna1g* and Balb3T3 cells to DNT-DT_A_. MC3T3-E1, MC3T3-E1/Δ*Cacna1g* (left), and Balb3T3 (right) cells with or without *Cacna1g* complementation were treated with DNT-DT_A_. Each bar represents the mean ± SEM (*n* = 3). *, *P < *0.0001 (compared with E1 [left] and Balb3T3 [right]). (D) Ectopic expression of *Cacna1g* v3 restores the sensitivity of MC3T3-E1/Δ*Cacna1g* (left) and Balb3T3 (right) cells to DNT. The cells were treated with DNT and subjected to SDS-PAGE, followed by immunoblotting. (E) Immunoprecipitation (IP) assay to detect interactions between Ca_V_3.1 and DNT. After treatment with DNT, MC3T3-E1/Δ*Cacna1g*/+*Cacna1g* v3 cells were subjected to an immunoprecipitation assay, followed by immunoblotting with anti-DNT antibody and anti-Ca_V_3.1 antibody. (F) Competitive inhibition of the cytotoxicity of DNT-DT_A_ with ProTx-I. MC3T3-E1 cells were treated with DNT-DT_A_ at the indicated concentrations in the presence of ProTx-I and subjected to a cytotoxicity assay. Each bar represents the mean ± SEM (*n* = 3). *, *P < *0.05; **, *P < *0.0001 (compared with 0 μM ProTx-I in each DNT-DT_A_ dose group).

*Cacna1g* (*CACNA1G* in humans) encodes the T-type voltage-gated Ca^2+^ channel Ca_V_3.1, which comprises four domains, each containing six transmembrane helices (Fig. 3A). The gene carries at least 12 (for mouse) and 24 (for human) alternative splice variants (24–26). We succeeded in cloning three distinct cDNAs of the splice variants of *Cacna1g*, which were tentatively designated variant 1 (v1), v2, and v3 (Fig. 3A and B). Ectopic expression of v3 but not v1 and v2 restored sensitivity to DNT and DNT-DT_A_ of Δ*Cacna1g* cells and intrinsically DNT-resistant Balb3T3 cells (Fig. 3C and D and Fig. S2A to C). Immunoprecipitation assays revealed the interaction between extracellularly added DNT and Ca_V_3.1 on the membrane of MC3T3-E1/Δ*Cacna1g/*+*Cacna1g* v3 cells (Fig. 3E and Fig. S2D). The cytotoxic effects of DNT-DT_A_ on MC3T3-E1 cells were reduced in the presence of ProTx-I (Fig. 3F), a spider toxin that specifically binds to Ca_V_3.1 (27). These results demonstrate that certain splice variants of Ca_V_3.1 serve as the receptor conferring DNT sensitivity to cells. In the present study, v3, but not v1 and v2, functioned as the receptor. As the alternative splice sites of v1, v2, and v3 were located in the first domain (domain 1 [D1]) (Δ5′E2 and Δ5′E8) and the second domain (D2) (ΔE14 and Δ5′E16) (Fig. 3A and B), these domains may be involved in the interaction with DNT.

10.1128/mBio.03146-19.4 FIG S2 Ca_V_3.1 serves as the receptor for DNT from B. pertussis and B. bronchiseptica. (A) Ectopic expression of *Cacna1g* in MC3T3-E1 and MC3T3-E1/Δ*Cacna1g* cells. The cytosolic and membrane fractions of MC3T3-E1, MC3T3-E1/Δ*Cacna1g*, and MC3T3-E1/Δ*Cacna1g*/+*Cacna1g* (variant 1 [v1], v2, and v3) cells were subjected to SDS-PAGE, followed by immunoblotting for Ca_V_3.1. Transferrin receptor (TFR) and β-tubulin were used as markers for the cell membrane and cytosol, respectively. (B) Fluorescence microscopy of MC3T3-E1/Δ*Cacna1g* cells treated with DNT from B. bronchiseptica. The cells were stained with rhodamine-labeled phalloidin to detect actin fibers. DNT-sensitive MC3T3-E1 and MC3T3-E1/Δ*Cacna1g*+*Cacna1g* v3, but not MC3T3-E1/Δ*Cacna1g*, cells exhibited an intensive organization of actin stress fibers through Rho activation by recombinant DNT of B. bronchiseptica. Bar, 20 μm. (C) Immunoblotting for the deamidation of Rho in MC3T3-E1, MC3T3-E1/Δ*Cacna1g*, and MC3T3-E1/Δ*Cacna1g/*+*Cacna1g* v3 cells. The cells were exposed to 50 ng/ml of recombinant DNT of B. bronchiseptica (b) or B. pertussis (p) for 16 h. (D) Immunoprecipitation assay to detect the interaction between Ca_V_3.1 and B. bronchiseptica (b) DNT and B. pertussis (p) DNT. After treatment with 1 μg/ml of DNT at 20°C for 2 h, MC3T3-E1/Δ*Cacna1g/*+*Cacna1g* v3 cells were subjected to an assay with anti-DNT antibody or normal IgG, followed by immunoblotting with anti-DNT antibody and anti-Ca_V_3.1 antibody. Download FIG S2, EPS file, 1.3 MB. Copyright © 2020 Teruya et al. 2020 Teruya et al. This content is distributed under the terms of the Creative Commons Attribution 4.0 International license.

### DNT is a neurotropic toxin.

**FIG 4:**
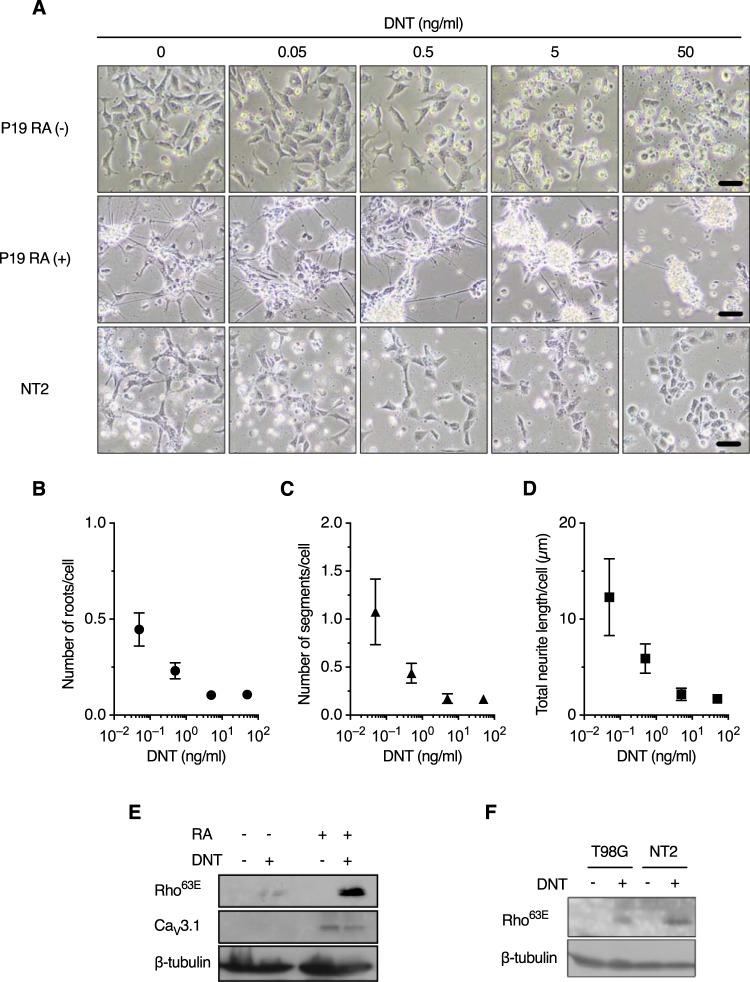
Neural cells are sensitive to DNT. (A) Morphological changes of P19 {undifferentiated [RA (−)] and differentiated [RA (+)]} and NT2 cells. The cells were treated with DNT at the indicated concentrations for 24 h. Bars, 50 μm. (B to D) Numbers of nerve roots and neurite segments and total length of neurites of P19 RA (+) cells (see also Fig. S3C in the supplemental material). The cells were treated with DNT at the indicated concentrations for 24 h in a 12-well plate, and the numbers of nerve roots (B) and neurite segments (C) and the total length of neurites (D) were evaluated from 4,000 to 25,000 cells in a single well using the Opera Phenix system and Harmony 4.5. Values are means ± SEM (*n* = 4). (E and F) Immunoblotting of P19 cells (E) and T98G and NT2 cells (F) for deamidated Rho. The cells were treated with 50 ng/ml of DNT for 24 h.

According to the public databases of the Genotype-Tissue Expression (GTEx) (https://www.gtexportal.org/home/) project (Table S4) and BioGPS (http://biogps.org/#goto=welcome), *CACNA1G* (human) and *Cacna1g* (mouse) are dominantly expressed in the cerebellum and other brain tissues and in female genital organs, suggesting that these tissues are targeted by DNT in *Bordetella* infection. We therefore examined if neural cells are affected by DNT. P19 murine embryonal carcinoma cells differentiate into neural cells, including neurons and glial cells, after incubation in the presence of retinoic acid (RA) (Fig. S3) (28, 29). The cells responded to DNT in both the differentiated and undifferentiated states, as judged by morphological alterations and the deamidation of Rho (Fig. 4A to E). Differentiated P19 cells highly expressed Ca_V_3.1, compared to undifferentiated cells, in which Ca_V_3.1 expression was barely detected by immunoblotting (Fig. 4E). Accordingly, Rho of the differentiated cells was highly deamidated. The differentiated cells lost neurites and aggregated upon treatment with DNT (Fig. 4A to D). These results are consistent with previous observations that neurite outgrowth was inhibited by the activation of the Rho signaling pathway (30–33). NTera2/cl.D1 (NT2) human embryonal carcinoma cells and T98G human glioblastoma cells were also sensitive to DNT, indicating that the toxin intoxicates cells of human origin, similarly to those of mouse origin (Fig. 4F).

10.1128/mBio.03146-19.5 FIG S3 Differentiation of P19 cells into neuronal cells and glial cells. The cells were allowed to differentiate into neural cells by incubation with retinoic acid (RA), as described previously (see reference 8 in Text S2). (A) After RA treatment, the cells were stained for the neuronal marker microtubule-assocated protein 2 (MAP2) (magenta) and the glial marker glial fibrillary acidic protein (GFAP) (green). Hoechst 33342 (blue) was used to stain the nuclei. Fluorescence images were collected using the Opera Phenix system (PerkinElmer). Bar, 50 μm. (B) The numbers of MAP2-positive cells and GFAP-positive cells were separately counted by using Harmony 4.5 (PerkinElmer), and the percentage of the respective cells compared to total cells (nuclei) is shown. Each bar represents the mean ± SEM (*n* = 4). (C) Definitions of roots, segments, and total length of neurites. Yellow circles in the left panel indicate the “roots” of neurites. Neurites branch out, as shown in the right panel. Each branch of neurites colored differently is a “segment.” “Total length” of neurites is the sum of the lengths of all segments. Download FIG S3, EPS file, 1.0 MB. Copyright © 2020 Teruya et al. 2020 Teruya et al. This content is distributed under the terms of the Creative Commons Attribution 4.0 International license.

**FIG 5:**
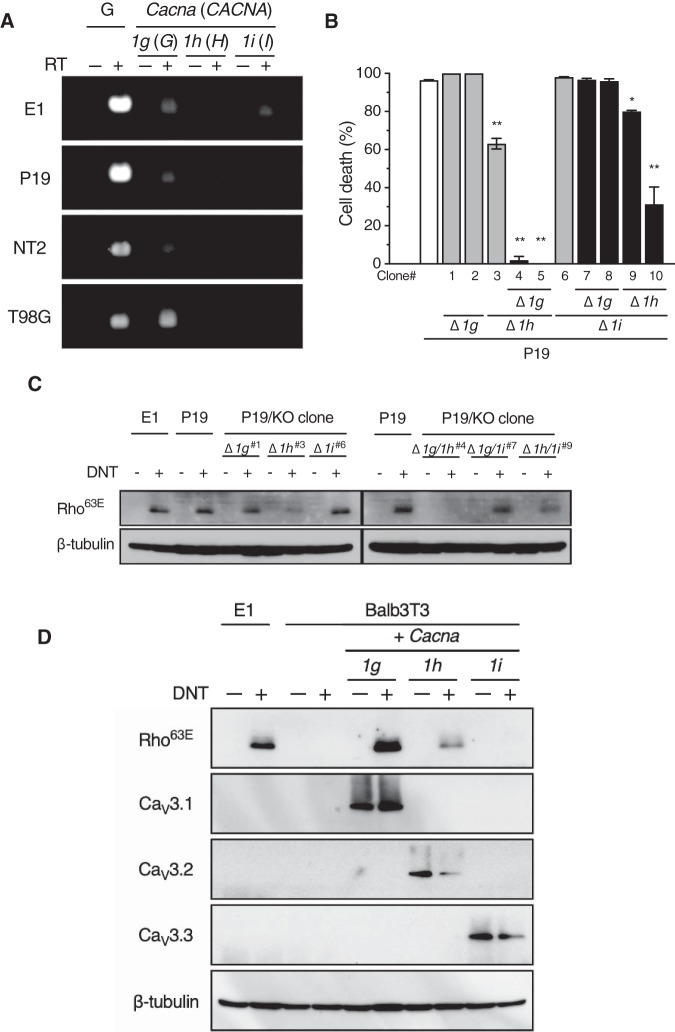
Sensitivity of isotypes of the calcium ion channels to DNT. (A) RT-PCR analyses of T-type Ca^2+^ channel transcripts (*Cacna1g*, *Cacna1h*, and *Cacna1i* for MC3T3-E1 and P19 cells of mouse origin and *CACNA1G*, *CACNA1H*, and *CACNA1I* for NT2 and T98G cells of human origin). RT, reverse transcription reaction; G, glyceraldehyde-3-phosphate dehydrogenase (*Gapdh* or *GAPDH*) (positive control). (B and C) Sensitivity of P19 cells deficient in *Cacna1g* (Δ*1g*), *Cacna1h* (Δ*1h*), and/or *Cacna1i* (Δ*1i*) to DNT-DT_A_ (B) and DNT (C). The cells were treated with DNT-DT_A_ or DNT and subjected to a cytotoxicity assay (B) or immunoblotting for deamidated Rho (C), respectively. Each bar represents the mean ± SEM (*n* = 3). *, *P < *0.05; **, *P < *0.0001 (versus parental P19 cells [B]). The numbers of selected clones in panel C correspond to those shown in panel B. KO, knockout. (D) DNT sensitivity of Balb3T3 cells expressing the isotypes of the T-type voltage-gated Ca^2+^ channels. The cells were treated with DNT and subjected to SDS-PAGE followed by immunoblotting for deamidated Rho (Rho^63E^), Ca_V_3.1, Ca_V_3.2, Ca_V_3.3, and β-tubulin.

In addition to Ca_V_3.1, Ca_V_3.2 and Ca_V_3.3 are known as the isotypes of the T-type voltage-gated Ca^2+^ channels, encoded by *CACNA1H* (*Cacna1h*) and *CACNA1I* (*Cacna1i*), respectively. As reverse transcription-PCR (RT-PCR) revealed that P19 cells express all the isotypes (Fig. 5A), we examined the sensitivity of the isotypes to the toxins using these cells (Fig. 5B and C). *Cacna1g* and *Cacna1h* double-knockout P19 cells were resistant to DNT-DT_A_ and DNT. The single and double knockouts of *Cacna1g* and/or *Cacna1i* did not abolish sensitivity. *Cacna1h* knockout cells, with or without *Cacna1i* knockout, exhibited moderate sensitivity to DNT-DT_A_ and DNT. In addition, Balb3T3 cells ectopically expressing Ca_V_3.1 and Ca_V_3.2, but not Ca_V_3.3, were sensitive to DNT (Fig. 5D). Taken together, Ca_V_3.1 (encoded by *Cacna1g*) and Ca_V_3.2 (*Cacna1h*), but not Ca_V_3.3 (*Cacna1i*), function as the receptors for DNT. As MC3T3-E1 cells do not express Ca_V_3.2 (Fig. 5A), we successfully identified *Cacna1g* as the receptor gene by the primary screening of sgRNA-introduced MC3T3-E1 cells with the CRISPR-Cas9 system.

### Neurological disorders caused by DNT in mice.

**FIG 6:**
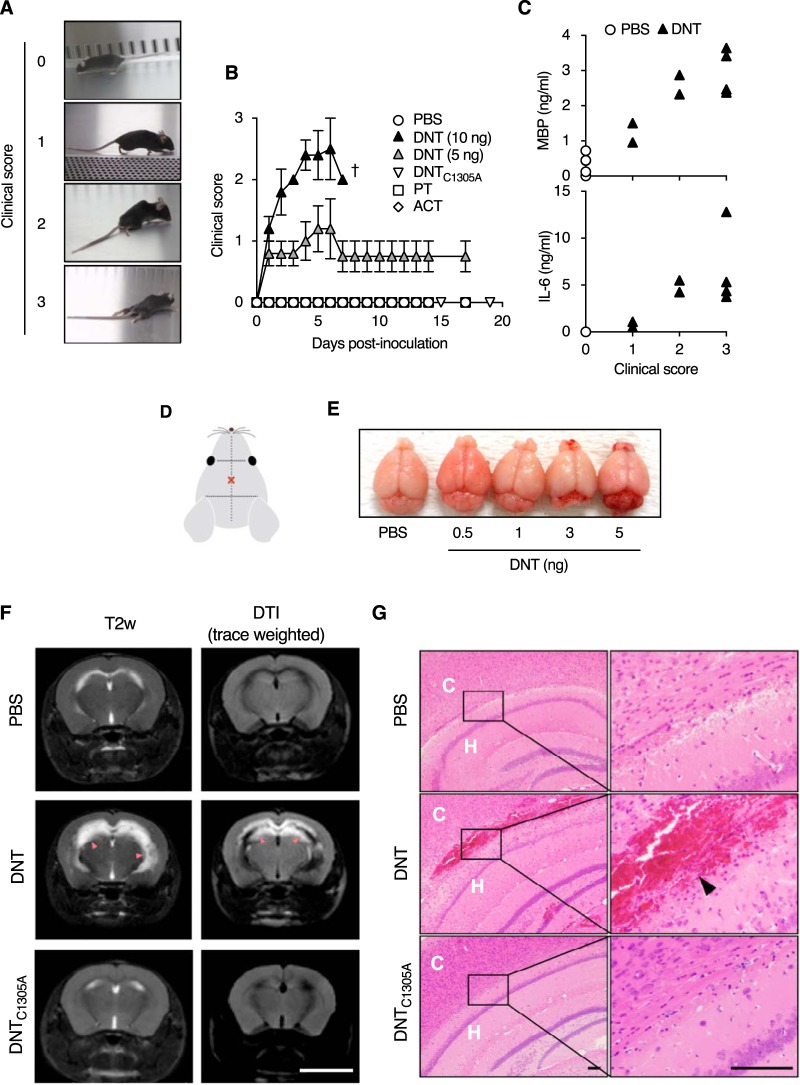
DNT-induced encephalitis in mice. (A and B) Clinical signs of neurological disorders in mice intracerebrally injected with DNT, DNT_C1305A_, PT, and ACT. The severities of signs were scored as follows: 0 for normal, 1 for limp tail and hind limb weakness, 2 for partial paralysis of hind limbs, and 3 for complete paralysis of hind limbs. Each plot represents means ± SEM (*n* = 5). †, all dead. One and two mice died 5 days and 13 days after injection with PT, respectively. (C) Concentrations of MBP (top) and IL-6 (bottom) in cerebrospinal fluids of mice intracerebrally injected with DNT (*n* = 8) or PBS (*n* = 5). Each data point, representing one mouse, was plotted on the ordinate against each clinical score on the abscissa. (D) Illustration indicating the injection site (×). Samples were injected with a needle 4 mm long and 0.4 mm in diameter. (E) Macroscopic images of mouse brains excised 3 days after DNT injection. Note the bloody spots around the cerebellum and olfactory bulb. (F) MRI images of brains of mice intracerebrally injected with 5 ng of DNT or DNT_C1305A_. The mice were subjected to MRI 3 days after injection. Bar, 5 mm. T2-weighted (T2w) imaging and diffusion tensor imaging (DTI) revealed abnormal high-intensity white signals (arrowheads) in the vicinity of cerebral ventricles of the DNT-injected mice, indicating severe inflammation (DTI) with watery infiltration (T2w). (G) Histological sections of brains of mice intracerebrally injected with 3 ng of DNT or 5 ng of DNT_C1305A_. Hemorrhage is noted in the specimen of a mouse injected with DNT (arrowhead). Bars, 100 μm. C, cerebral cortex; H, hippocampus.

The above-described results suggest that DNT is a neurotropic toxin. Because Ca_V_3.1 is dominantly expressed in the central nervous system (Table S4), we examined if DNT causes any neurological disorders by intracerebrally injecting the toxin into mice. From 1 day after injection, the mice developed neurological symptoms such as flaccidity of tails and hind limbs (Fig. 6A and B and Movie S1). These symptoms were not observed with 10-fold-larger amounts of PT or ACT (Fig. 6B). An enzymatically inactive mutant of DNT, DNT_C1305A_ (14), did not cause any symptoms, indicating that the Rho-targeting transglutaminase activity of the toxin is necessary for its neurotoxicity. In the mice injected with DNT, myelin basic protein (MBP) and interleukin-6 (IL-6) levels in the cerebrospinal fluid (CSF) were markedly increased (Fig. 6C), indicating demyelination and inflammation in the central nervous system. These signs were similarly stated in a recent case report of pertussis-associated encephalitis/encephalopathy (3). The levels of both factors correlated with the severity of clinical symptoms. Hemorrhage was noted in brains of mice injected with DNT upon both macroscopic and microscopic examinations (Fig. 6D, E, and G). Magnetic resonance imaging revealed severe inflammation with watery infiltration along the cerebroventricular area in DNT-injected mice (Fig. 6F). From these results, we concluded that DNT, when intracerebrally injected, causes encephalitis in mice.

10.1128/mBio.03146-19.10 MOVIE S1 Clinical signs of mice intracerebrally injected with DNT. Download Movie S1, MOV file, 13.9 MB. Copyright © 2020 Teruya et al. 2020 Teruya et al. This content is distributed under the terms of the Creative Commons Attribution 4.0 International license.

## DISCUSSION

DNT is commonly produced by pathogenic *Bordetella* species such as B. pertussis, B. parapertussis, and B. bronchiseptica. B. bronchiseptica DNT is known to cause turbinate atrophy in swine atrophic rhinitis, B. bronchiseptica infection, by inhibiting osteoblastic differentiation (18–20). It was also reported that the toxin directly affects osteoblastic MC3T3-E1 cells, suggesting that osteoblastic cells express the DNT receptor. However, as only a few cell lines were found to be sensitive to DNT (20, 21, 23), the toxin receptor was considered not to be ubiquitous and instead was considered to be unique to particular cells. In this study, we demonstrated that the T-type voltage-gated Ca^2+^ channels Ca_V_3.1 and Ca_V_3.2 serve as DNT receptors and confer sensitivity to the toxin. Ca_V_3.1 is reportedly expressed in osteoblastic cells during osteogenesis (34). This is consistent with previous results that DNT affects osteoblastic cells. Indeed, the sensitivity of osteoblastic MC3T3-E1 cells to the toxin was found to be dependent on the expression of Ca_V_3.1 (Fig. 5A).

It is also known that the Ca_V_3 channels, which are expressed in neural tissues and cardiac and smooth muscles, are involved in neuronal excitability, pacemaker activity in the sinoatrial node, the circadian rhythm of sleep and wakefulness, hormone secretion, and peripheral pain sensation (35–37). According to public databases, Ca_V_3.1 is dominantly expressed in the central nervous system, whereas Ca_V_3.2 expression does not exhibit a characteristic tissue distribution (https://www.gtexportal.org/home/gene/CACNA1H). Being attracted to this point, we examined the neurotoxicity of DNT at the cellular level and found that it affected neural cells *in vitro*, indicating that DNT has aspects of a neurotropic toxin; this is the first example of a neurotropic virulence factor produced by B. pertussis. These results prompted us to explore if DNT is involved in neurological disorders that are recognized as pertussis encephalitis/encephalopathy; peripheral nervous disorders have not been observed in B. pertussis infection, although the Ca_V_3 channels are also known to be distributed in the peripheral nervous system. The intracerebral injection of DNT, but not PT and ACT, caused encephalitis in mice.

**FIG 7:**
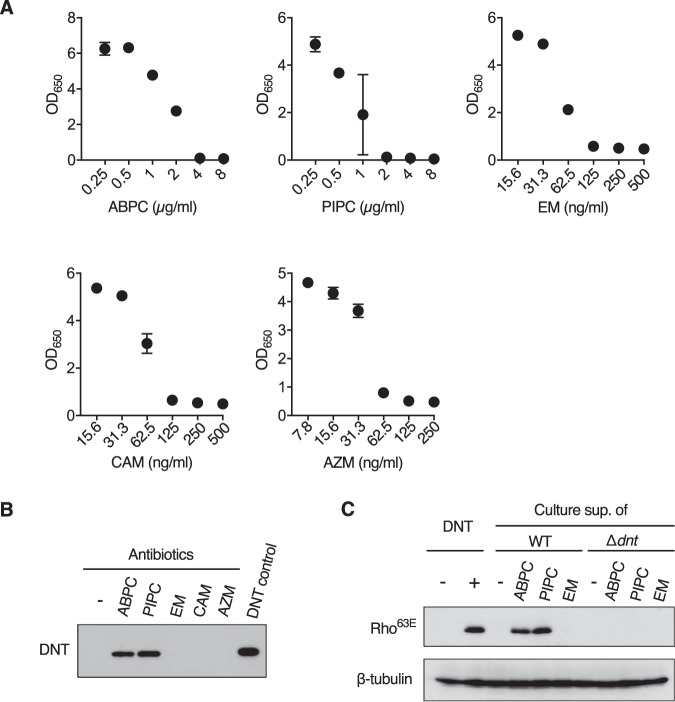
Liberation of DNT from B. pertussis treated with antibiotics. (A) Dose-response curve of bactericidal effects of antibiotics on B. pertussis. B. pertussis Tohama I was suspended in SS medium to give an optical density at 650 nm (OD_650_) value of 0.2. After a 6-h incubation, the indicated antibiotics were added to the cultures at the indicated concentrations. After further incubation for 24 h, OD_650_ values of the cultures were measured. ABPC, ampicillin; PIPC, piperacillin; EM, erythromycin; CAM, clarithromycin; AZM, azithromycin. Each plot represents means ± SEM (*n* = 3). No error bars appear for some points because the SEM values were smaller than the size of the symbols. (B) Immunoblotting for DNT in the culture supernatants of B. pertussis treated with the following antibiotics: ABPC at 4 μg/ml, PIPC at 2 μg/ml, EM at 0.125 μg/ml, CAM at 0.125 μg/ml, and AZM at 0.125 μg/ml. The concentrations of antibiotics were determined according to the dose-response curve shown in panel A. (C) Immunoblotting for deamidated Rho in MC3T3-E1 cells treated with the culture supernatants or DNT. The cells were treated with the culture supernatants of B. pertussis wild-type (WT) or Δ*dnt* cells that were incubated with or without the antibiotics.

In B. pertussis infection, encephalitis/encephalopathy (here, we use the term “pertussis encephalopathy” according to previous reports) has long been recognized as a rare complication, which develops in up to 1% of patients and imposes a significant burden (6, 7); studies from 2001 to 2003 in the United States reported that 33 of 28,998 patients (0.11%) developed encephalopathy (38). The pathophysiology and etiological agents of encephalopathy remain unknown. Possible explanations included hemorrhage in the central nervous system resulting from increased venous pressure due to coughing paroxysms, hypoxia, venous stasis attributable to leukocytosis, hypoglycemia, and secondary infections by neurotropic microbes. However, some case reports negated these explanations and instead pointed out unknown toxins or toxin-like components of B. pertussis as the causative agents (3, 4, 7). DNT, which causes encephalitis in mice, may be the most probable candidate for such a bacterial component. On the other hand, DNT has been considered to play little role in the pathogenesis of pertussis, partly because it is not secreted but remains localized in the bacterial cytoplasm (8, 39, 40). To bridge this gap, we retrieved case reports of pertussis encephalopathy that clearly stated the course of the disease (1–5, 7). Only a few reports were available, but in 4 of 6 case reports, the patients, in the early stages or before developing neurological disorders, were administered β-lactam antibiotics including, cefuroxime (4), amoxicillin (5), cephalexin and ampicillin (7), or piperacillin (3). β-Lactam antibiotics inhibit the synthesis of peptidoglycan and may therefore liberate DNT from bacterial cells. Indeed, we confirmed that treatment of B. pertussis infection with ampicillin and piperacillin resulted in the release of active DNT (Fig. 7). Macrolides such as erythromycin, clarithromycin, and azithromycin, which are the first-line antibiotics for pertussis, did not release DNT.

In this study, we raised the possibility of DNT as an etiological agent of pertussis encephalopathy. However, as not all pertussis patients develop encephalopathy, several other factors should be involved in its development. The use of β-lactam antibiotics releasing DNT from bacterial cells may be one of the risk factors. Cofactors may also be required to help DNT cross the blood-brain barrier and enter the central nervous system. PT, which was reported to affect the integrity of cerebral barriers (41, 42), may function as such an accessory factor. Indeed, PT is known to exacerbate experimental autoimmune encephalomyelitis in mice (43, 44). In addition, the anamnestic history, vaccinations, age, and sex of patients and other bacterial components may influence the onset of encephalopathy. Although further studies are required to address many remaining questions, our study provides a clue to understanding the pathophysiology of pertussis encephalopathy.

## MATERIALS AND METHODS

Antibodies, immunoprecipitation assays, magnetic resonance imaging (MRI) analyses, and other utilized methods are given in Text S1 in the supplemental material.

10.1128/mBio.03146-19.1 TEXT S1 Supplemental materials and methods. Download Text S1, DOCX file, 0.01 MB. Copyright © 2020 Teruya et al. 2020 Teruya et al. This content is distributed under the terms of the Creative Commons Attribution 4.0 International license.

10.1128/mBio.03146-19.2 TEXT S2 References related to the supplemental material. Download Text S2, DOCX file, 0.1 MB. Copyright © 2020 Teruya et al. 2020 Teruya et al. This content is distributed under the terms of the Creative Commons Attribution 4.0 International license.

### Bacterial strains and cultures.

B. pertussis strain Tohama I was maintained in the laboratory. A *dnt*-deficient mutant of B. pertussis Tohama I was generated according to methods described previously (45). B. pertussis was grown in Stainer-Scholte (SS) medium or on Bordet-Gengou agar (Becton, Dickinson, Franklin Lakes, NJ, USA) containing 0.4% (wt/vol) polypeptone or HiPolypeptone (Wako Pure Chemical Industries, Ltd., Japan), 0.8% glycerol, 20% defibrinated horse blood, and 10 μg/ml ceftibuten (BG plate). The culture supernatants of B. pertussis were harvested by centrifugation at 6,800 × *g* for 5 min and filtered through a 0.22-μm-pore-size filter.

### Recombinant DNT and DNT derivatives.

The primers used for plasmid construction are listed in Table S1. The plasmids encoding DNT and DNT derivatives of B. bronchiseptica were constructed as follows. pQEDNTwt and pGEXDNT_1–54_-His were constructed as described previously (22). pDTA1 was provided by E. Mekada, Osaka University (46).

10.1128/mBio.03146-19.6 TABLE S1 Primers used in this study. Download Table S1, DOCX file, 0.01 MB. Copyright © 2020 Teruya et al. 2020 Teruya et al. This content is distributed under the terms of the Creative Commons Attribution 4.0 International license.

For DNT-DT_A_/pQE40, DNA fragments were amplified by PCR with a combination of primers, EcoRV-DNT_1009_Fw and DNT_1185_-DTARv, and pQEDNTwt as the template and with another combination of primers, HindIII-DTA_28_Rv and DNT_1185_-DTAFw, and pDTA1 as the template. The resultant fragments were used as the templates for PCR to amplify DNA fragments encoding DNT-DT_A_ using the primers EcoRV-DNT_1009_Fw and HindIII-DTA_28_Rv. The obtained fragments were inserted into the EcoRV-HindIII site of pQEDNTwt by the seamless ligation cloning extract (SLiCE) technique (47).

For DNT-DT_A_/pColdII, the DNA fragment encoding DNT-DT_A_ was amplified by PCR with a combination of primers, NdeI-DNT_2_Fw and EcoRI-DTA_218_Rv, and DNT-DT_A_/pQE40 as the template and inserted into the NdeI-EcoRI site of pColdII (TaKaRa) by the SLiCE technique.

The plasmids for DNT and DNT derivatives of B. pertussis were constructed as follows. For BpDNTwt/pQE40, a DNA fragment covering the DNT gene was amplified by nested PCR using B. pertussis Tohama I genomic DNA as the template with the first primers BpDNT-nested-F and BpDNT-nested-R and the second primers BamHI-BpDNT_2_-F and Bp-DNT_1464_-HindIII-R. The resultant PCR product was inserted into the BamHI-HindIII site of pQE40 (Qiagen) by the SLiCE technique.

For BpDNT_C1305A_/pQE40, site-directed mutagenesis was used to replace Cys with Ala at position 1305 in BpDNTwt/pQE40 using a QuikChange kit and the primer pair DNT_C1350A_-F and DNT_C1305A_-R.

The expression plasmids were introduced into Escherichia coli M15(pREP4) or BL21(DE3), and the recombinant DNT proteins were produced and purified by Ni or glutathione affinity chromatography according to the manufacturer’s instructions (Qiagen, TaKaRa, and GE Healthcare). The recombinant proteins of full-length DNT were further purified by anion-exchange chromatography with a MonoQ column (GE Healthcare) in 20 mM Tris-HCl (pH 7.6) containing 1 M urea and eluted with a linear gradient of NaCl from 10 to 500 mM in the same buffer. The purified DNT proteins were dialyzed against and kept in 50 mM phosphate buffer (pH 7.4) containing 0.3 M Na_2_SO_4_ and 1 M urea at 4°C.

### Cell cultures.

All media for cell cultures were supplemented with 10% fetal calf serum (FCS) unless otherwise specified. All cell lines were grown at 37°C under 5% CO_2_ in air. The MC3T3-E1 (mouse osteoblast) and T98G (human glioblastoma) cell lines, provided by K. Irie, Fukuoka University, were cultured in alpha minimum essential medium (Gibco Laboratories). The Balb3T3 (clone A31) (mouse embryo fibroblast), Vero (African green monkey kidney epithelial), COS7 (African green monkey kidney fibroblast), and Rat-1 (rat fibroblast) cell lines were maintained in Dulbecco’s modified Eagle’s medium (DMEM) (Sigma-Aldrich). P19 (mouse embryonal carcinoma) cells were cultured in alpha minimum essential medium (Sigma-Aldrich) containing 1% GlutaMAX supplement (catalog number 35050-061; Gibco Laboratories). Differentiation of P19 cells was stimulated by incubation in the presence of retinoic acid (RA) at 500 nM as described previously (28). NTera2/cl.D1 (NT2) (human embryonal carcinoma) cells were cultured in DMEM plus GlutaMAX-I (catalog number 10566-016; Gibco Laboratories). CHO-K1 (Chinese hamster ovary epithelial) cells were grown in Ham’s F-12 medium (Wako). 293FT (human embryo kidney epithelial) and Plat-E (human embryo kidney epithelial) cells were cultured according to the manufacturer’s instructions.

### Cas9-expressing MC3T3-E1 cells.

The DNA fragment encoding enhanced green fluorescent protein (EGFP) was obtained from pX330mEGFP (48) by digestion with FseI and inserted into the FseI site of pPB-pgkBSD-CBh-hSpCas9n, which is a derivative of pPB-SA hyg NP21 pA (48, 49), yielding pPB-pgkBSD-CBh-hSpCas9n-EGFP. Subsequently, the pgkBSD region of the plasmid was replaced by pgkNeo, a phosphoglycerate kinase 1 (PGK) promoter-driven neomycin resistance gene. The obtained plasmid was designated pPB-pgkNeo-CBh-hSpCas9n-EGFP. MC3T3-E1 cells were cotransfected with pPB-pgkNeo-CBh-hSpCas9n-EGFP and pCMV-hyPBase (49), which carries a *piggyBac* transposase, using the Neon transfection system (Invitrogen) according to the manufacturer’s instructions and were cultured for 24 h and then for 6 days in the presence of 400 μg/ml of G418. Independent clones of the surviving cells were isolated by the limiting-dilution method. A clone that highly expressed Cas9-EGFP, as judged by fluorescence microscopy, was selected and designated Cas9-E1.

### Generation of the genome-wide Cas9-E1-sgRNA library and screening.

We utilized genome-wide mouse lentiviral CRISPR guide RNA (gRNA) library v1 (50) (catalog number 50947; Addgene), which contains five unique sgRNAs for each of 19,150 genes. 293FT cells were transfected with the library plasmids by using Lipofectamine 2000 (Invitrogen) according to the manufacturer’s instructions. The medium was replaced 24 h after transfection, and lentiviral vectors were obtained 2 days after transfection by centrifugation of the culture supernatant at 1,700 × *g* for 15 min at 4°C. At a multiplicity of infection (MOI) of 0.3, 10^7^ cells of Cas9-E1 were infected with the lentiviral library on 150-mm dishes in the presence of 8 μg/ml of Polybrene (Millipore) such that a single sgRNA was introduced into more than 30 cells. The cells were selected by incubation in the presence of 8 μg/ml of puromycin and screened with DNT-DT_A_, as follows.

For the first round of screening, 5 × 10^7^ cells were treated with 2 μg/ml of DNT-DT_A_ in 100-mm dishes. After the 36-h treatment, the cells were washed three times with Dulbecco’s modified phosphate-buffered saline (D-PBS) to remove dead cells. The remaining cells were reseeded, incubated for 24 h, and subjected to another round of toxin screening. After three rounds of screening, the remaining cells were collected, and their genomic DNA was extracted by isopropanol precipitation and subjected to the following analyses, together with the genomic DNA from untreated original cells. The regions of sgRNA were amplified by PCR from the genomic DNA templates using the primers pST-Cas9-S27 and pST-Cas9-AS28 with Q5 Hot Start high-fidelity DNA polymerase (New England BioLabs). The PCR products were sequenced using Ion PGM (Thermo Fisher Scientific).

### Generation of MC3T3-E1 and P19 knockout cells.

pX330mEGFP carrying sgRNA for each target gene (Table S2) at the BbsI site was introduced into MC3T3-E1 cells by electroporation or into P19 cells with Lipofectamine 3000 (Invitrogen). After the 48-h incubation, EGFP-expressing cells were sorted and placed into each well of a 96-well plate using the FACSAria II system (BD Biosciences). The knockout cells were used for the following assays after cultivation for at least 14 days.

10.1128/mBio.03146-19.7 TABLE S2 sgRNA used in this study. Download Table S2, DOCX file, 0.01 MB. Copyright © 2020 Teruya et al. 2020 Teruya et al. This content is distributed under the terms of the Creative Commons Attribution 4.0 International license.

10.1128/mBio.03146-19.8 TABLE S3 Genes identified after DNT-DT_A_ screening. Download Table S3, DOCX file, 0.01 MB. Copyright © 2020 Teruya et al. 2020 Teruya et al. This content is distributed under the terms of the Creative Commons Attribution 4.0 International license.

10.1128/mBio.03146-19.9 TABLE S4 Gene expression for *CACNA1G*. Download Table S4, DOCX file, 0.01 MB. Copyright © 2020 Teruya et al. 2020 Teruya et al. This content is distributed under the terms of the Creative Commons Attribution 4.0 International license.

### Establishment of *Cacna*-expressing cells.

cDNA of *Cacna1g* was obtained by PCR using the cDNA library prepared from MC3T3-E1 cells as the template and primers BS300 and BS297 and inserted into the BamHI-NotI site of pCX4pur, provided by Tsuyoshi Akagi (51). Plat-E cells were transfected with the resultant plasmids, pCX4pur-*Cacna1g*-v1, -v2, and -v3, using Lipofectamine 3000 according to the manufacturer’s instructions. The medium was replaced 16 h after transfection. The culture supernatant containing viral vectors was collected by centrifugation 2 days after transfection and filtered on a 0.45-μm-pore-size polyvinylidene difluoride membrane. Balb3T3 or *Cacna1g* knockout MC3T3-E1 cells (2 × 10^4^ cells) were seeded into each well of a 6-well plate. After 2 days, the medium was replaced with that containing the appropriately diluted retroviral supernatant and 8 μg/ml of Polybrene. Two days after infection, the puromycin-resistant cells were selected by incubation with 8 μg/ml of puromycin for at least 3 days. cDNAs of *Cacna1h* and *Cacna1i* were obtained by PCR using Fantom clones (52) (clone identification numbers M5C1001I06 and M5C1084D17, respectively; DNAFORM) as the templates, the primers BamHI-*Cacna1h*-F1 and *Cacna1h*-NotI-R1, and the primers BamHI-*Cacna1i*-F1 and *Cacna1i*-NotI-R1. The cDNAs were cloned into the vector and introduced into Balb3T3 cells as described above.

### Cell culture assay.

For the assay of DNT-DT_A_ cytotoxicity, 5 × 10^3^ cells were seeded into each well of a 96-well plate and incubated for 24 h. The medium was replaced with that containing DNT-DT_A_, and the cells were subsequently incubated for 36 h or the indicated periods. DNT-DT_A_ was used at 2 μg/ml unless otherwise specified. Cell viability was quantified using Cell Counting kit 8 (catalog number 343-07623; Wako). The absorbance at 450 nm of each well was measured using the Glomax multidetection system (Promega). The rate of cell death was calculated by the equation cell death (%) = (*A*_buf_ − *A*_tox_)/(*A*_buf_ − *A*_trtn_) × 100, where *A*_buf_, *A*_tox_, and *A*_trtn_ are the net absorbances of the untreated sample, the toxin-treated sample, and the Triton X-100-treated sample, respectively.

For the detection of cytopathic effects by DNT, cells were seeded into a 24-well plate at 2.0 × 10^4^ cells/well and incubated for 24 h. The cells were further incubated without FCS for 24 h and then incubated with or without 50 ng/ml of DNT for an additional 16 h. The cells were fixed with 4% paraformaldehyde in D-PBS, permeabilized with 0.5% Triton X-100 in D-PBS for 5 min, and stained with rhodamine-phalloidin. Images were taken with a FluoView FV10i microscope (Olympus, Tokyo, Japan). Independently, cells were seeded at 2.4 × 10^5^ cells/well into a 6-well plate and treated with DNT as described above. After treatment, the cell lysates were obtained by ultrasonic treatment using a Bioruptor sonicator (Cosmo Bio, Tokyo, Japan), followed by centrifugation. Proteins in the supernatants were precipitated with cold 10% trichloroacetic acid and subjected to SDS-PAGE and immunoblotting for deamidated Rho.

P19 cells were differentiated into neural cells according to a method reported previously (28). The differentiated cells were seeded into a 6-well plate at 3.6 × 10^6^ cells/well, incubated for 6 days, and treated with DNT preparations at the indicated concentrations. Undifferentiated cells were seeded at 1.2 × 10^5^ cells/well and similarly treated with the DNT preparations after a 2-day incubation.

### Animal experiments.

Seven-week-old female specific-pathogen-free (SPF) C57BL/6J mice (Japan SLC) were intracerebrally inoculated with 25 μl of DNT (10 or 5 ng [ca. 60 or 30 fmol]), DNT_C1305A_ (10 ng [ca. 60 fmol]), pertussis toxin (PT) (65 ng [ca. 600 fmol]), or adenylate cyclase toxin (ACT) (110 ng [ca. 600 fmol]) under anesthesia with isoflurane or a mixture of midazolam, medetomidine, and butorphanol at final doses of 2, 0.3, and 5 mg/kg body weight, respectively (Fig. 6D), and monitored for clinical signs of encephalopathy 1 min a day for up to 19 days. In independent experiments, cerebrospinal fluid (CSF) was obtained from the cisterna magna 7 days after inoculation, and the concentrations of myelin basic protein and interleukin-6 in CSF were determined by using an MBP enzyme-linked immunosorbent assay (ELISA) kit (catalog number OKCD02716; Aviva Systems Biology) and a mouse IL-6 DuoSet ELISA (catalog number DY406-05; R&D Systems), respectively.

For histological studies, mice were intracerebrally injected with PBS or recombinant DNT (3 ng) or DNT_C1305A_ (5 ng) of B. pertussis, sacrificed by CO_2_ after 3 days, and perfused with 0.9% NaCl followed by 4% paraformaldehyde (Wako) through the right atrium. The brain was excised, fixed in 4% paraformaldehyde at 4°C overnight, and embedded in paraffin using the TP120 tissue processor (Thermo Fisher Scientific). Thin sections were obtained using a horizontal microtome (Yamato Kohki, Saitama, Japan) with an 8-μm setting and stained with hematoxylin and eosin (HE). Images were taken with an FSX-100 fluorescence microscope (Olympus, Osaka, Japan).

All animal experiments were approved by the Animal Care and Use Committee of the Research Institute for Microbial Disease, Osaka University, and carried out according to the regulations on animal experiments at Osaka University.

### Statistical analysis.

Statistical analyses were performed by one-way analysis of variance and Dunnett’s or Tukey’s multiple-comparison test using Prism 8 (GraphPad Software).
