# Supplementary material for: Bordetella Dermonecrotic Toxin Is a Neurotropic Virulence Factor That Uses CaV3.1 as the Cell Surface Receptor
Source: mBio. 2020 Mar 24;11(2):e03146-19. doi: 10.1128/mBio.03146-19 (PMC7157530; doi:10.1128/mBio.03146-19)
Supplement: TEXT S1 [file mBio.03146-19-s0001.docx]

**Text S1: Supplemental Materials and Methods**

**Antibodies.** Mouse monoclonal and rabbit polyclonal antibodies against DNT were prepared as reported previously (1, 2). The anti-Rho^63E^ polyclonal antibody was prepared as previously described (3). The polyclonal antibody against DT was provided by E. Mekada. The following antibodies were purchased from the indicated vendors: anti-His tag (Sigma Aldrich, #H1029), anti-β-tubulin (Wako, #014-25041), anti-transferrin receptor (Zymed, #13-6800), anti-MAP2 (Sigma Aldrich, #M9942), anti-GFAP (Dako, #A0334), anti-Ca_V_3.1 (Millipore, #AB5491), anti-Ca_V_3.2 (alomone labs, #ACC-025), anti-Ca_V_3.3 (alomone labs, #ACC-009), HRP-conjugated goat anti-rabbit IgG (Jackson, #111-035-144), HRP-conjugated goat anti-mouse IgG (Jackson, #115-035-062), Alexa568-conjugated goat anti-mouse IgG (Invitrogen, #A11031), and Alexa488-conjugated goat anti-rabbit IgG (Invitrogen, #A11034).

**Immunoprecipitation assay.** Confluent MC3T3-E1/Δ*Cacna1g/*+*Cacna1g* v3 cells in a 100-mm dish were treated with 1 µg/ml of DNT at 20°C for 2 h, washed with cold wash buffer (20 mM HEPES, pH 7.4, containing 150 mM NaCl), and lysed with 1 ml of the lysis buffer (50 mM Tris-HCl, pH 7.6, containing 1% Nonidet P-40, 0.5% sodium deoxycholate, and 1% Protease Inhibitor Cocktail (Nacalai, #25955)). The resultant lysate was centrifuged at 12,000 × g for 10 min at 4˚C, and 500 µl of the supernatant was treated with 1 µg of anti-DNT polyclonal antibody for 2 h at 4˚C with gentle shaking. Separately, Protein G Sepharose 4 Fast Flow (GE Healthcare) was washed twice with H_2_O and once with the lysis buffer, and suspended in the lysis buffer containing 5 µg/ml of anti-rabbit IgG antibody. The lysate treated with anti-DNT antibody was mixed with 20 µl of the protein G gel beads, and incubated for 2 h at 4˚C. The protein G beads were collected by centrifugation, washed five times with the lysis buffer, and boiled in two-fold concentrated SDS sample buffer (24 mM Tris-HCl, 0.8% SDS, 4% glycerol, and 50 mM dithiothreitol with bromophenol blue). After centrifugation, the supernatants were subjected to SDS-PAGE followed by immunoblotting.

**MRI analyses.** MRI data were acquired using the BioSpec 117/11 AvanceIII system with ^1^H QD coil and Para Vision software (Bruker). T2-weighted and diffusion-tensor imaging (DTI) conditions were as follows: RARE (rapid acquisition with relaxation enhancement) method was used for T2-weighted imaging with RARE Factor 8 and effective TE=48 msec, TR=3000 msec, number of average 4 times. The DitStandard method was used for DTI imaging, TE=18 msec, TR=3250 msec, SW Tensor images were acquired in 6 directions with one A0 image; the B value was 1000 s/mm^2^. The FOV was 2.6 cm, the slice thickness was 1 mm, and matrix was 200 for T2-weighted and 128 for DTI, and then processed to 256 for further analysis. DTI images were processed using the DTI reconstruction software. The mice were anesthetized by sevoflurane during image acquisition.

**Treatment of *B. pertussis* with antibiotics.** *B. pertussis* Tohama I was treated with ampicillin (ABPC, 4 µg/ml), piperacillin (PIPC, 2 µg/ml), erythromycin (EM, 0.125 µg/ml), clarithromycin (CAM, 0.125 µg/ml), or azithromycin (AZM, 0.125 µg/ml) for 24 h, and each culture supernatant was applied to SDS-PAGE and subsequent immunoblotting for DNT.

**Others.** The cytosolic and membrane fractions of cells were obtained as described previously (4). ProTx-I was purchased from Peptide Institute, Inc. RT-PCR was carried out as follows: Total RNA was extracted from cells with TRIzol (Thermo Fisher Scientific). Reverse transcription was carried out with 1 µg of total RNA and the Primescript RT reagent kit (TaKaRa) according to the manufacturer’s instructions, followed by PCR with the primers listed in Table S1 under the following conditions: initial denaturation for 2 min at 94˚C and 35 cycles of 98˚C for 10 sec and 68˚C for 30 sec. PT was purified from *B. pertussis* culture as previously reported (5). Recombinant ACT was obtained by the method described previously (4). The protein concentration of test materials used in this study was determined using BCA Protein Assay Reagents (Thermo Scientific) according to the manufacturer’s instructions. For immunoblotting, the samples were electrically transferred onto polyvinylidene difluoride membranes (Millipore) after SDS-PAGE. The membranes were then treated with 10% skim milk, and the transferred proteins were probed with appropriate antibodies and visualized with Immobilon Western Chemiluminescent HRP substrate (Millipore).

**Data availability.** Raw sequencing data are provided on requests to the corresponding author on reasonable request. All other data generated during the current study are included in this published article.
