# Supplementary material for: Bordetella Dermonecrotic Toxin Is a Neurotropic Virulence Factor That Uses CaV3.1 as the Cell Surface Receptor
Source: mBio. 2020 Mar 24;11(2):e03146-19. doi: 10.1128/mBio.03146-19 (PMC7157530; doi:10.1128/mBio.03146-19)
Supplement: TEXT S2 [file mBio.03146-19-s0002.docx]

**Text S2: References related to supplemental material.**

1. **Horiguchi Y**, **Nakai T**, **Kume K**. 1990. Simplified procedure for purification of *Bordetella bronchiseptica* dermonecrotic toxin. FEMS Microbiol Lett **66**:39–43.

2. **Matsuzawa T**, **Kashimoto T**, **Katahira J**, **Horiguchi Y**. 2002. Identification of a receptor-binding domain of *Bordetella* dermonecrotic toxin. Infect Immun **70**:3427–3432.

3. **Horiguchi Y**, **Inoue N**, **Masuda M**, **Kashimoto T**, **Katahira J**, **Sugimoto N**, **Matsuda M**. 1997. *Bordetella bronchiseptica* dermonecrotizing toxin induces reorganization of actin stress fibers through deamidation of Gln-63 of the GTP-binding protein Rho. Proc Natl Acad Sci USA **94**:11623–11626.

4. **Fukui-Miyazaki A**, **Toshima H**, **Hiramatsu Y**, **Okada K**, **Nakamura K**, **Ishigaki K**, **Shinzawa N**, **Abe H**, **Horiguchi Y**. 2018. The Eukaryotic Host Factor 14-3-3 Inactivates Adenylate Cyclase Toxins of *Bordetella bronchiseptica* and *B. parapertussis*, but not *B. pertussis*. mBio **9**:49–15.

5. **Skelton SK**, **Wong KH**. 1990. Simple, efficient purification of filamentous hemagglutinin and pertussis toxin from *Bordetella pertussis* by hydrophobic and affinity interaction. J Clin Microbiol **28**:1062–1065.

6. **Horiguchi Y**. 2012. Swine Atrophic Rhinitis Caused by *Pasteurella multocida* Toxin and *Bordetella* Dermonecrotic Toxin, pp. 113–129. *In* Aktories, K, Orth, JHC, Adler, B (eds.), Current Topics in Microbiology and Immunology. Springer Berlin Heidelberg, Berlin, Heidelberg.

7. **Fukui-Miyazaki A**, **Ohnishi S**, **Kamitani S**, **Abe H**, **Horiguchi Y**. 2011. *Bordetella* dermonecrotic toxin binds to target cells via the N-terminal 30 amino acids. Microbiology and Immunology **55**:154–159.

8. **Tsukamoto K**, **Ozeki C**, **Kohda T**, **Tsuji T**. 2015. CRISPR/Cas9-Mediated Genomic Deletion of the Beta-1, 4 N-acetylgalactosaminyltransferase 1 Gene in Murine P19 Embryonal Carcinoma Cells Results in Low Sensitivity to Botulinum Neurotoxin Type C. PLoS ONE **10**:e0132363.
