# Supplementary material for: Bordetella Dermonecrotic Toxin Is a Neurotropic Virulence Factor That Uses CaV3.1 as the Cell Surface Receptor
Source: mBio. 2020 Mar 24;11(2):e03146-19. doi: 10.1128/mBio.03146-19 (PMC7157530; doi:10.1128/mBio.03146-19)
Supplement: TABLE S1 [file mBio.03146-19-st001.docx]

**Table S1: Primers used in this study**

| Primer | Sequence (5’-3’) | For construction of |
| --- | --- | --- |
| *Eco*RV-DNT_1009_Fw | GGCAATGTGTTCGATATCTCGA | DNT-DT_A_/pQE40 |
| DNT_1185_-DTARv | TCAACAACATCATCAGCGCCGGAAATATCCCCATGGTTGA |  |
| *Hin*dIII-DTA_218_Rv | TCAGCTAATTAAGCTCTATCGCCGTACGCGATTTC |  |
| DNT_1185_-DTAFw | TCAACCATGGGGATATTTCCGGCGCTGATGATGTTGTTGA |  |
| *Nde*I-DNT_2_Fw | ATCATCATCATCATATGGATAAAGATGAATCGGCATTGCG | DNT-DT_A_/pColdII |
| *Eco*RI-DTA_218_Rv | CGACAAGCTTGAATTCCTATCGCCGTAACGCGATTTC |  |
| BpDNT-nested-F | GTGTGTTTGCTAGCCTGACTAACAGG | BpDNTwt/pQE40 |
| BpDNT-nested-R | CAATAAAGGCCGCGTCCCAATGC |  |
| *Bam*HI-BpDNT_2_-F | TCACCATCACGGATCCGATAAAGATGAATCGGCATTGC |  |
| BpDNT_1464_-*Hin*dIII-R | TCAGCTAATTAAGCTTTCAGACCGGCGCCGGAAAC |  |
| DNT_C1305A_-F | GGCTCCTTGAGCGGGGCCACGACGATGGTTGGG | BpDNT_C1305A_/pQE40 |
| DNT_C1305A_-R | CCCAACCATCGTCGTGGCCCCGCTCAAGGAGCC |  |
| pST-Cas9-S27 | CTTGAAAGTATTTCGATTTCTTGG | Amplification of sgRNA regions in the Cas9-E1-sgRNA library |
| pST-Cas9-AS28 | ACTCGGTGCCACTTTTTCAA |  |
| BS300 | CTAGACTGCCGGATCCACCATGGATGAGGAAGAGGATGGAGCG | pCX4pur-*Cacna1g*-v1, v2, v3 |
| BS297 | ATTGTTACCGCGGCCGCTCAGGGATCCAGGTCTGTTGGG |  |
| *Bam*HI-*Cacna1h*-F1 | CTAGACTGCCGGATCCACCATGACCGAGGGCACGC | pCX4pur-*Cacna1h* |
| *Cacna1h*-*Not*I-R1 | ATTGTTACCGCGGCCGCCTACACAGGCTCATCTCCACTGTC |  |
| *Bam*HI-*Cacna1i*-F1 | CTAGACTGCCGGATCCACCATGGCTGACAGCAACTTACCACCCTC | pCX4pur-*Cacna1i* |
| *Cacna1i*-*Not*I-R1 | ATTGTTACCGCGGCCGCTCATCTCTTCCTTTTGCTCGCCGCATCC |  |
| h*GAPDH*_RT-PCR_F1 | AGGGCTGCTTTTAACTCTGGT | RT-PCR for *GAPDH* |
| h*GAPDH*_RT-PCR_R1 | CCCCACTTGATTTTGGAGGGA |  |
| h*CACNA1G*_RT-PCR_F2 | GCTCTTTGGAGACCTGGAGTGT | RT-PCR for *CACNA1G* |
| h*CACNA1G*_RT-PCR_R2 | TAGGCGAGATGACCGTGTTG |  |
| h*CACNA1H*_RT-PCR_F1 | TTGGGTTCCGTCGGTTCT | RT-PCR for *CACNA1H* |
| h*CACNA1H*_RT-PCR_R1 | ATGCCCGTAGCCATCTTCA |  |
| h*CACNA1I*_RT-PCR_F1 | ATCGGTTATGCTTGGATTGTCA | RT-PCR for *CACNA1I* |
| h*CACNA1I*_RT-PCR_R1 | TGCTCCCGTTGCTTGGTCTC |  |
| *G3PDH* F | ACCACAGTCCATGCCATCAC | RT-PCR for *Gapdh* |
| *G3PDH* R | TCCACCACCCTGTTGCTGTA |  |
| m*Cacna1g*_RT-PCR_F1 | GGAGCTGGAGCTAGAGATGA | RT-PCR for *Cacna1g* |
| m*Cacna1g*_RT-PCR_R1 | CAGACAAGATGGAGCCTGACT |  |
| m*Cacna1h*_RT-PCR_F1 | TCTCTGAGCCTCTCACGGAT | RT-PCR for *Cacna1h* |
| m*Cacna1h*_RT-PCR_R1 | GATGTGGCTGACCTCCTCAT |  |
| m*Cacna1i*_RT-PCR_F1 | CTGGAGACCTGGATGAATGCT | RT-PCR for *Cacna1i* |
| m*Cacna1i*_RT-PCR_R1 | CAAGAGGGTGCAGTTGACACT |  |
