# Supplementary material for: Bordetella Dermonecrotic Toxin Is a Neurotropic Virulence Factor That Uses CaV3.1 as the Cell Surface Receptor
Source: mBio. 2020 Mar 24;11(2):e03146-19. doi: 10.1128/mBio.03146-19 (PMC7157530; doi:10.1128/mBio.03146-19)
Supplement: TABLE S2 [file mBio.03146-19-st002.docx]

**Table S2: sgRNAs used in this study**

| Target gene | Sequence (5’-3’) |
| --- | --- |
| *Dhx29* | GAAACATCCCGGTACGTAGG |
| *Taok1* | GGTCACTTACCTATGGATCA |
| *Cacna1g* | GTCGGCCGTCGCCGTCTGTG |
| *Tmem151b* | GCGCTTCTTCGCCGAGAACG |
| *Nrg2* | GTCCTTGAACCAGCGATAGG |
| *Cacna1g* (for P19 cells) | GCCTCATAGTCCAGACCGCA |
| *Cacna1h* | GACTACAACGTGTGCCGCTC |
| *Cacna1i* | GAGGCCAAGCGCCGTGCTCT |
