# Supplementary material for: Bordetella Dermonecrotic Toxin Is a Neurotropic Virulence Factor That Uses CaV3.1 as the Cell Surface Receptor
Source: mBio. 2020 Mar 24;11(2):e03146-19. doi: 10.1128/mBio.03146-19 (PMC7157530; doi:10.1128/mBio.03146-19)
Supplement: TABLE S3 [file mBio.03146-19-st003.docx]

**Table S3: Genes identified after the DNT-DT_A_ screening**

| Gene name | sgRNA | | | Protein name |
| --- | --- | --- | --- | --- |
|  | Total reads | All*^a^* | Hit*^b^* |  |
| *Dhx29* | 27136 | 5 | 5 | ATP-dependent RNA helicase DHX29 |
| *Taok1* | 4868 | 5 | 5 | Serine/threonine-protein kinase TAO1 |
| *Cacna1g* | 3050 | 5 | 5 | Voltage-dependent T-type calcium channel subunit alpha-1G |
| *Nf2* | 269730 | 5 | 4 | Merlin |
| *Tada1* | 8452 | 5 | 4 | Transcriptional adapter 1 |
| *Eif4g2* | 7528 | 5 | 4 | Eukaryotic translation initiation factor 4 gamma 2 |
| *Taf5l* | 5792 | 5 | 4 | TAF5-like RNA polymerase II p300/CBP-associated factor-associated factor 65 kDa subunit 5L |
| *Wdr85* | 2567 | 5 | 4 | Diphthine methyltransferase |
| *Stk40* | 39483 | 5 | 3 | Serine/threonine-protein kinase 40 |
| *Trp53* | 35435 | 5 | 3 | Cellular tumor antigen p53 |
| *Taf6l* | 27853 | 5 | 3 | TAF6-like RNA polymerase II p300/CBP-associated factor-associated factor 65 kDa subunit 6L |
| *Fam48a* | 9589 | 4 | 3 | Transcription factor SPT20 homolog |
| *Tada2b* | 4350 | 5 | 3 | Transcriptional adapter 2-beta |
| *Tmem151b* | 3014 | 5 | 3 | Transmembrane protein 151B |
| *Zfp867* | 2680 | 5 | 3 | Zinc finger protein 867 |
| *Sergef* | 2183 | 5 | 3 | Secretion-regulating guanine nucleotide exchange factor |
| *Nrg2* | 1628 | 5 | 3 | Pro-neuregulin-2, membrane-bound isoform |
| *Prkar1a* | 1544 | 5 | 3 | cAMP-dependent protein kinase type I-alpha regulatory subunit |
| *Ccdc101* | 1170 | 5 | 3 | SAGA-associated factor 29 |
| *Rfwd2* | 642 | 3 | 3 | E3 ubiquitin-protein ligase COP1 |
| *Fam198b* | 11823 | 5 | 2 | Golgi-associated kinase 1B |
| *Fam120b* | 9802 | 5 | 2 | Constitutive coactivator of peroxisome proliferator-activated receptor gamma |
| *Map1lc3a* | 9605 | 5 | 2 | Microtubule-associated proteins 1A/1B light chain 3A |
| *Epha10* | 9584 | 5 | 2 | Ephrin type-A receptor 10 |
| *Zfp69* | 7938 | 5 | 2 | Zinc finger protein 69 |
| *Ralgapb* | 3244 | 5 | 2 | Ral GTPase-activating protein subunit beta |
| *Cnga4* | 2397 | 5 | 2 | Cyclic nucleotide-gated cation channel alpha-4 |
| *Dph1* | 1852 | 3 | 2 | 2- (3-amino-3-carboxypropyl) histidine synthase subunit 1 |
| *Map4k4* | 1570 | 5 | 2 | Mitogen-activated protein kinase kinase kinase kinase 4 |
| *Cep135* | 1425 | 5 | 2 | Centrosomal protein of 135 kDa |
| *Atp4b* | 1343 | 5 | 2 | Potassium-transporting ATPase subunit beta |

*^a^* The number of unique sgRNAs for each gene in the original library.

*^b^* The number of unique sgRNAs detected after screening.
