# Supplementary material for: Bordetella Dermonecrotic Toxin Is a Neurotropic Virulence Factor That Uses CaV3.1 as the Cell Surface Receptor
Source: mBio. 2020 Mar 24;11(2):e03146-19. doi: 10.1128/mBio.03146-19 (PMC7157530; doi:10.1128/mBio.03146-19)
Supplement: TABLE S4 [file mBio.03146-19-st004.docx]

**Table S4: Gene expression for *CACNA1G* (ENSG00000006283.13)*^a^***

| Tissue | Median TPM*^b^* | n*^c^* |
| --- | --- | --- |
| Brain - Cerebellum | 34.76 | 173 |
| Cervix - Ectocervix | 18.92 | 6 |
| Cervix - Endocervix | 12.56 | 5 |
| Brain - Cortex | 12.04 | 158 |
| Brain - Cerebellar Hemisphere | 10.44 | 136 |
| Brain - Frontal Cortex (BA9) | 9.530 | 129 |
| Brain - Hypothalamus | 7.570 | 121 |
| Ovary | 7.130 | 133 |
| Vagina | 6.390 | 115 |
| Fallopian Tube | 5.620 | 7 |
| Brain - Anterior cingulate cortex (BA24) | 4.620 | 121 |
| Brain - Substantia nigra | 3.850 | 88 |
| Uterus | 2.920 | 111 |
| Brain - Amygdala | 2.310 | 100 |
| Heart - Atrial Appendage | 2.160 | 297 |
| Adipose - Visceral (Omentum) | 1.940 | 355 |
| Prostate | 1.755 | 152 |
| Skin - Not Sun exposed (Suprapubic) | 1.370 | 387 |
| Brain - Caudate (Basal ganglia) | 1.290 | 160 |
| Brain - Hippocampus | 1.230 | 123 |
| Testis | 1.230 | 259 |
| Brain - Nucleus accumbens (Basal ganglia) | 1.220 | 147 |
| Brain - Spinal cord (Cervical c-1) | 1.220 | 91 |
| Brain - Putamen (Basal ganglia) | 1.175 | 124 |
| Skin - Sun exposed (Lower leg) | 1.150 | 473 |
| Breast - Mammary Tissue | 1.090 | 290 |

***^a^*** Data of GTEx Analysis Release V7 (https://www.gtexportal.org/home/gene/CACNA1G, dbGaP Accession phs000424.v7.p2) are tabulated.

*^b^*TPM: Transcripts per million

*^c^*n: Number of samples
